# Association between Long-Term Change in Arterial Stiffness and Cardiovascular Outcomes in Kidney Transplant Recipients: Insights from the TRANSARTE Study

**DOI:** 10.3390/jcm11051410

**Published:** 2022-03-04

**Authors:** Madonna Salib, Arnaud Simon, Nicolas Girerd, Anna Kearney-Schwartz, Patrick Rossignol, Athanase Benetos, Luc Frimat, Sophie Girerd

**Affiliations:** 1 Centre d’Investigations Cliniques-1433, Université de Lorraine, Inserm U1116, CHRU Nancy, F-CRIN INI-CRCT, 54000 Nancy, France; m.salib@chru-nancy.fr (M.S.); n.girerd@chru-nancy.fr (N.G.); p.rossignol@chru-nancy.fr (P.R.); 2 Nephrology Department, University Hospital of Nancy, 54500 Vandoeuvre les Nancy, France; a.simon@chru-nancy.fr (A.S.); l.frimat@chru-nancy.fr (L.F.); 3 Défaillance Cardiovasculaire Aigüe et Chronique (DCAC), Université de Lorraine, Inserm, 54000 Nancy, France; a.kearney-schwartz@chru-nancy.fr (A.K.-S.); a.benetos@chru-nancy.fr (A.B.); 4 Department of Geriatrics, Federation Hospital-University on Cardiovascular Aging (FHU-CARTAGE), University Hospital of Nancy, Université de Lorraine, 54000 Nancy, France

**Keywords:** arterial stiffness, cardiovascular diseases, kidney transplantation, pulse-wave velocity

## Abstract

(1) Background: Increased arterial stiffness is associated with cardiovascular (CV) diseases in end-stage renal disease (ESRD) patients, and CV mortality remains higher in kidney transplantation (KT) recipients compared to in the general population. KT is associated with an improvement in arterial stiffness in the early post-transplant period, followed by a potential re-worsening in the late period. In a cohort of KT patients, we evaluated the associations of pulse-wave velocity (PWV) measured at different time-points (pre-transplant, and early and late post-transplant periods) with CV morbi-mortality, as well as the evolution between these measurements with CV morbi-mortality. (2) Methods: Forty KT recipients with a 10-year follow-up were included. The association of PWV with CV events was assessed with multivariable cox analysis. Backward linear regressions were conducted to identify the determinants of PWV at 1 year and those of the long-term evolution of PWV after KT (delta PWV at 1 year—latest PWV). (3) Results: The absence of arterial stiffening during the long-term follow-up after KT is associated with a lower CV outcome rate (HR for the delta PWV = 0.76 (0.58–0.98), *p* = 0.036). Age at KT is associated with the worsening of arterial stiffness in the late post-transplantation period (β for the delta PWV = −0.104, *p* = 0.031). A high PWV at 1 year was associated with a potential for recovery during follow-up (β = 0.744, *p* < 0.0001). (4) Conclusions: The absence of PWV worsening in the late post-transplantation period was significantly associated with a lower risk of CV events, whereas early changes in PWV were not. Finding an intervention capable of reducing long-term PWV could improve the prognosis of KT recipients.

## 1. Introduction

Cardiovascular (CV) diseases are the leading cause of death in patients with end-stage renal disease (ESRD). After kidney transplantation (KT) and the restoration of renal function, the risk of CV mortality remains high compared with the general population [1]. Increased PWV, a marker of arterial stiffness, has been reported to be a strong risk factor of CV morbi-mortality in ESRD [2]. In KT, some studies have also reported this association [3,4].

The mechanisms underlying arterial stiffness in KT are numerous, involving the pretransplant history of patients (the duration of ESRD or dialysis, and co-morbidities such as hypertension or diabetes). Age also remains a key determinant of PWV in KT [5]. Nevertheless, data on the evolution of PWV suggest a decrease in PWV during the first year after transplantation [6,7] followed by an increase in PWV after that point [1]. This long-term deterioration of PWV may indicate that factors arising with transplantation, e.g., immunosuppressive drugs, could have a detrimental impact on PWV [8]. It was also reported that that donor age was an independent factor for PWV in KT with a deceased donor [9].

Therefore, considering the potential improvement of arterial stiffness in the early post-transplantation period [6,7]—and potential re-worsening in the late-transplantation period [1]—we aimed to evaluate the associations between CV morbi-mortality and PWV measured at different time-points (pre-transplantation, early post-transplantation, and late post-transplantation periods), as well as the evolution between these measurements with CV morbi-mortality, in a cohort of kidney transplant patients. In this framework, we investigated the association of arterial stiffness with CV events in KT recipients from the monocentric TRANSARTE study (Transplantation and Arteries).

## 2. Materials and Methods

### 2.1. Study Population

A detailed description of the TRANSARTE study has been published previously [10]. Briefly, 100 ESRD patients were recruited from the waiting list of the University Hospital of Nancy to characterize the evolution of arterial stiffness after KT in a population of ESRD patients. Among the kidney-transplanted patients, the present study was carried out on 40 renal transplant recipients with available predefined PWV measurements, as previously described [10]. The composite primary outcome of the study is a CV event (defined by acute coronary syndrome, myocardial infarction, angioplasty, vascular angioplasty, coronary bypass, ischemic or hemorrhagic stroke, angioplasty of lower extremity arteries, amputation, and atrial fibrillation).

### 2.2. Measurement of PWV

PWV measurements were performed using the Complior^®^ technique (ALAM Medical, Saint Quentin Fallavier, France), which simultaneously records arterial pulse waves at carotid and femoral sites. The propagation time (t) of the incident wave was measured through mechanotransducer probes, the carotid–femoral distance (D) was evaluated with a tape measure, and PWV (m/s) was obtained by the formula PWV = D × 0.8/(t).

### 2.3. Statistical Analysis

Analyses were performed using R version 4.0.2 (R Development Core Team, Vienna, Austria). The means are presented with SD and median, with 25th and 75th percentiles. The *p*-values are considered significant if they are <0.05. The association of PWV with CV events was assessed using Cox models. Backward linear regression was performed to identify the determinants of PWV at 1 year, and absolute changes in PWV post-transplantation (delta between PWV before transplantation and at 1-year, and between 1- and 4-years post-transplantation). Gender, age at transplantation; mean arterial blood pressure (MBP); hypercholesterolemia; diabetes mellitus; smoking; history of angina or myocardial infarction; previous strokes; CNI (Tacrolimus/Cyclosporine); and cause of ESRD were considered as independent variables. In addition, PWV at one-year post-transplantation was considered in the model focusing on late change in PWV. All variables with a *p* < 0.15 in univariable analysis were used as candidate variables in the multivariable model prior to backward selection procedures.

## 3. Results

### 3.1. Baseline Characteristics of Patients

In the 40 patients receiving either a living (*n* = 6) or a deceased (*n* = 34) donor allograft, the median age at transplantation (years) was 53 [47–60]. 19 (47.5%) were female. Of the subjects, 5 (12.5%) were diabetic, 2 (5.0%) had history of angina or myocardial infarction, and 2 (5.0%) had previously experienced strokes. The mean pre-transplantation, 1-year post-transplantation and latest post-transplantation PWVs (m/s) (respectively, measured at a median delay of 7 [2–11] months before KT, 13 [11–25] months after KT, and 52 [37–62] months after KT) were 8.9 ± 2.45, 9.0 ± 2.8, and 8.9 ± 2.4 (m/s), respectively (*p* = 0.96). The majority of patients received calcineurin inhibitors (CNI) (95%), most of them cyclosporine (55.3%). During the median follow-up period of 10.9 years, 12 patients died, 11 patients had a CV event and 6 patients returned to dialysis (Table 1).

### 3.2. Association of PWV with CV Events

**Figure 1:**
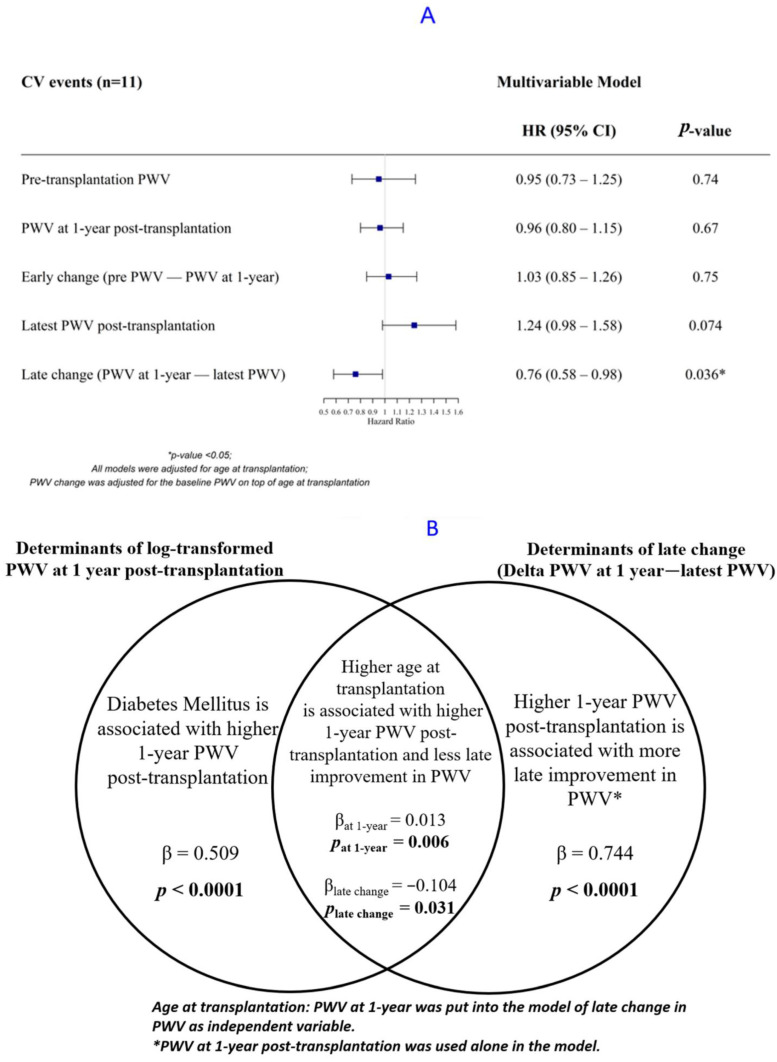
(**A**) Forest plot of the association between pulse-wave velocity (PWV) and cardiovascular (CV) events; (**B**) the determinants of PWV using backward linear regression.

In multivariable analysis adjusted for PWV post-transplantation at 1 year, and age at transplantation, the absence of arterial worsening in the late post-transplantation period was significantly associated with a lower risk of CV events (HR for the delta PWV at 1 year—latest PWV = 0.76 (0.58–0.98)). On the contrary, the evolution of PWV between the pre-transplant period and the early post-transplantation period, as well as the PWV at 1 year, were not significantly associated with CV morbi-mortality (Figure 1). The complete output of the Cox models is provided in Supplementary Table S1.

### 3.3. Determinants of PWV Using Backward Linear Regression

The backward multivariate regression analyses identified that age at KT and diabetes were significantly associated with an increased arterial stiffness at 1 year post-transplantation (β = 0.013, *p* = 0.0 06 and β = 0.509, *p* < 0.0001, respectively). Age at KT was also associated with the worsening of arterial stiffness in the late post-transplantation period, even after adjusting for PWV at 1 year (β for the delta (PWV at 1 year—PWV at 4 years = −0.104, *p* = 0.031)). A higher PWV at 1-year was associated with a PWV decrease over the late post-transplantation period (β = 0.744, *p* < 0.0001) (Figure 1). Importantly, MBP was not retained as a significant factor in the selection process. A detailed description of the backward regression model is provided in Supplementary Table S2.

## 4. Discussion

Despite the improvement of renal function conferred by KT, CV mortality remains higher in KT recipients compared with the general population. In KT, some studies demonstrated that an increased arterial stiffness was associated with CV mortality [3,4]. Nevertheless, the evolution of arterial stiffness is complex after KT; some studies reported an early improvement [6,7], and others reported a long-term re-worsening [1]. Vascular remodeling is complex and involves alterations of the extracellular matrix by uremic toxins, which are not fully reversed by KT [7]. Moreover, immunosuppressive therapy is associated with hypertension, diabetes and dyslipidemia, and may indirectly or directly impact the vascular system.

The present study focuses on the association between CV morbi-mortality and the PWV measured at different time-points, as well as the evolution of the PWV through time. Notably, despite the low number of patients, we had, for the first time, the opportunity to measure the arterial stiffness in the pre-KT, the early post-KT, and the late post-KT periods. Moreover, we analyzed CV outcomes after a ten-year follow-up period.

In a study on 220 kidney transplant recipients, carotid–femoral PWV evaluated at 3 and 12 months after KT was significantly associated with morbi-mortality 5.5 years after KT, whereas the delta of PWV between month 12 and month 3 was not [11]. However, the present study added that an increased PWV between month 12 and month 48 is associated with CV events (adjusted for PWV at 1-year after KT and age at KT) in kidney recipients 10 years after KT. This suggests an impact of non-traditional factors in the late post-KT period, probably related to immunosuppressive therapy.

Unsurprisingly, age is associated with increased PWV at 1 year, but also with less improvement in the long-term post-transplantation period. Ignace et al. previously reported that older patients had a higher improvement in PWV in the early post-transplantation period at 3 months post-KT [5]. We can hypothesize that despite this early improvement, arteries from older patients are more prone to deterioration related to immunosuppressive regimens after a long-term exposition.

On the contrary, increased PWV at 1 year is associated with increased potential improvement in the long-term post-transplantation period. One can hypothesize that arterial stiffness mainly related to CKD, but not age, has a greater potential for recovery after KT, and that the susceptibility of the vascular system to immunosuppressive therapy is less pronounced among the youngest patients with ESRD.

It is worth mentioning that, similarly to the results of Kolonko et al. [12], a history of diabetes mellitus was a significant determinant of PWV 1 year after transplantation in the present study. Notably, diabetes mellitus was not related to the worsening of the PWV change post-transplantation, suggesting the stability of PWV in diabetic patients after 1 year of KT. Indeed, it was evident that patients with greater age, diabetes mellitus and higher PWV at baseline had an increased PWV 1 year after transplantation [13,14].

## 5. Limitations

This study is a single-center study with a small sample size. This prevented the use of more advanced statistical analyses such as splines to determine the most adequate prognostic PWV thresholds, or the addition of some possibly important covariates in the multivariable analysis. However, we assessed ESRD-related arterial stiffness over a long period of time in a peculiar population of kidney-transplanted patients, something that has not been reported previously. Further long-term studies should be conducted to better ascertain the long-term changes in PWV following KT.

There was no preemptive KT in this cohort. Therefore, it was not possible to include the residual renal function before KT to assess its potential impact on the evolution of arterial stiffness after KT.

## 6. Conclusions

In this analysis, the absence of PWV- worsening in the late post-transplantation period was significantly associated with a lower risk of CV events. In contrast, the change in PWV observed early during the post-transplantation period was not associated with CV events.

In summary, the present study suggests that the worsening of aortic stiffness in the late post-KT period has a negative impact on long-term CV morbi-mortality. Ultimately, finding an intervention able to reduce PWV could improve the prognosis of KT recipients.

## Figures and Tables

**Table 1:** Baseline characteristics and outcomes of patients.

	Overall Population *N* = 40
Baseline characteristics	
Female (%)	19 (47.5)
Age at transplantation (years)	53 [47–60]
Medical history	
Hypercholesterolemia (%)	20 (50.0)
Smoking (%)	16 (40.0)
Diabetes (%)	5 (12.5)
History of angina or myocardial infarction (%)	2 (5.0)
Previous Strokes (%)	2 (5.0)
Cause of ESRD (%)	
Diabetes	2 (5.0)
Glomerulopathy	7 (17.5)
Malformation	8 (20.0)
Polycystic kidney disease	10 (25.0)
Vascular	6 (15.0)
Other	7 (17.5)
Pre-transplantation dialysis (%)	30 (75.0)
Hemodialysis (%)	23 (76.7)
Peritoneal dialysis (%)	7 (23.3)
Dialysis duration (years)	2.7 [2.1–3.5]
Transplantation characteristics	
Calcineurin inhibitors (%)	38 (95.0)
Cyclosporine (%)	21 (55.3)
Tacrolimus (%)	17 (44.7)
Donor type	
Living donor (%)	6 (15.0)
Deceased donor (%)	34 (85.0)
Standard-criteria donors (SCD)	17 (51.5)
Expanded-criteria donors (ECD)	16 (48.5)
Arterial stiffness characteristics	
Duration between pre-transplantation PWV and transplantation (months)	7 [2–11]
Pre-transplantation PWV (m/s)	8.9 ± 2.5
Pre-transplantation mean arterial blood pressure (mmHg)	101 ± 14
Duration between transplantation and 1-year post-transplantation PWV (months)	13 [11–25]
1-year post-transplantation PWV (m/s)	9.0 ± 2.8
1-year post-transplantation mean arterial blood pressure (mmHg)	94 ± 12
Duration between transplantation and the latest post-transplantation PWV (months)	52 [37–62]
The latest post-transplantation PWV (m/s)	8.9 ± 2.4
The latest post-transplantation mean arterial blood pressure (mmHg)	91 ± 12
Post-transplantation characteristics	
eGFR at 1 year	48.5 [40.5–54.3]
eGFR at the last contact	
All patients	48.0 [25.8–53.8]
Not back to dialysis patients	48.5 [42.3–56.8]
Follow-up time (years)	10.9 [8.2–12.0]
Back to dialysis or preemptive re-transplantation (%)	6 (15.0)
Death (%)	12 (30.0)
CV events post-transplantation (%)	11 (27.5)

Data presented as mean ± standard deviation; number (percent); and median [interquartile range]. Abbreviations: ESRD: end-stage renal disease; PWV: pulse-wave velocity; eGFR: estimated glomerular filtration rate (mL/min/1.73 m^2^); CV: cardiovascular. CV events post-transplantation, the composite primary outcome of the study, is defined by acute coronary syndrome, myocardial infarction, angioplasty, vascular angioplasty, coronary bypass, ischemic or hemorrhagic stroke, angioplasty of lower extremity arteries, amputation, and atrial fibrillation.

## Data Availability

The data presented in this study can be made available upon reasonable request to the corresponding author.

## Supplementary Materials

The following supporting information can be downloaded at: https://www.mdpi.com/article/10.3390/jcm11051410/s1, Table S1: Association of pulse-wave velocity (PWV) with cardiovascular (CV) events; Table S2: Determinants of absolute changes in pulse-wave velocity (PWV) post-transplantation (PWV at 1 year—latest PWV) using backward linear regression.
