# Supplementary material for: Association between Long-Term Change in Arterial Stiffness and Cardiovascular Outcomes in Kidney Transplant Recipients: Insights from the TRANSARTE Study
Source: J Clin Med. 2022 Mar 4;11(5):1410. doi: 10.3390/jcm11051410 (PMC8911013; doi:10.3390/jcm11051410)
Supplement: Supplementary file 1 [file jcm-11-01410-s001.zip › jcm-1594657-supplementary.pdf]

Supplementary Materials

Supplementary Table S1: Association of pulse wave velocity (PWV) with cardiovascular (CV) events

CV events (n=11)	Univariable HR (95% CI)	p-value	Multivariable ** HR (95% CI)	p-value
Pre-transplantation PWV	1.00 (0.77 - 1.31)	0.98	0.95 (0.73 - 1.25)	0.74
PWV at 1-year post-transplantation	1.01 (0.85 - 1.18)	0.95	0.96 (0.80 - 1.15)	0.67
Early change (pre PWV – PWV at 1-year) *	0.99 (0.82 - 1.20)	0.96	1.03 (0.85 - 1.26)	0.75
Latest PWV post-transplantation	1.34 (1.08 - 1.65)	**0.008**	1.24 (0.98 - 1.58)	**0.074**
Late change (PWV at 1- year – latest PWV) *	0.71 (0.55 - 0.90)	**0.006**	0.76 (0.58 - 0.98)	**0.036**

**models of PWV change were adjusted for the baseline PWV*
*** All models were adjusted for age at transplantation*
Results with p value less than 5% were emphasized using bold letters

Supplementary Table S2: Determinants of absolute changes in pulse wave velocity (PWV) post-transplantation (PWV at 1-year – latest PWV) using backward linear regression

Late PWV change (m/s)	Univariable*				Multivariable (backward selection) *			
	ß	(95% CI)		p-value	ß	(95% CI)		p-value
		Lower	Upper			Lower	Upper	
First PWV post-transplantation (m/s) **	0.662	0.405	0.918	**<0.0001**	0.744	0.489	1.000	**<0.0001**
Sex (1=Male, 2=Female)	1.125	-0.313	2.563	**0.12**	–	–	–	–
Age at transplantation (years)	-0.104	-0.198	-0.010	**0.031**	-0.104	-0.198	-0.010	**0.031**
Mean arterial blood pressure (mmHg)	-0.028	-0.095	0.033	0.35				
Hypercholesterolemia (1=Yes, 0=No)	0.532	-0.952	2.015	0.47				
Diabetes (1=Yes, 0=No)	1.565	-1.405	4.536	0.29				
Smoking (1=Yes, 0=No)	-0.828	-2.291	0.634	0.26				
History of angina or myocardial infarction (1=Yes, 0=No)	1.207	-2.331	4.746	0.49				
Previous strokes (1=Yes, 0=No)	-1.924	-5.202	1.355	0.24				
CNI (1=Tacrolimus, 2= Ciclosporin)	0.085	-1.549	0.719	0.92				
**Cause of ESRD**				**0.11**	–	–	–	–
Glomerulopathy	0.478	-3.895	4.852	0.83				
Malformation	1.023	-3.415	5.462	0.64				
Polycystic kidney disease	-0.146	-4.402	4.11	0.94				
Vascular	0.674	-3.826	5.101	0.77				
Other	0.800	-3.251	4.852	0.69				

**Univariable and multivariable models are adjusted on first PWV post-transplantation*
*** In this model PWV was used alone in the model*
Mean arterial blood pressure is at the moment of the latest PWV; CNI, Calcineurin inhibitors; ESRD, end-stage renal disease (Reference = diabetes)
All variables with a p <0.15 in univariable analysis were used as candidate variables in the multivariable model prior to backward selection procedures.
